# Poly[[triaqua­[μ_4_-*N*-(4-carboxyl­ato­phenyl)­iminodiacetato]sodium(I)zinc(II)] dihydrate]

**DOI:** 10.1107/S1600536808036441

**Published:** 2008-11-13

**Authors:** Dong-Sheng Ma

**Affiliations:** a College of Chemistry and Materials Science, Heilongjiang University, Harbin 150080, People’s Republic of China

## Abstract

In the title coordination polymer, {[NaZn(C_11_H_8_NO_6_)(H_2_O)_3_]·2H_2_O}_*n*_, the Zn atom is coordinated in a distorted tetra­hedral environment by three carboxyl­ate O atoms from two (4-carboxyl­atophenyl­imino)diacetate ligands and one water mol­ecule; the Na atom is in an distorted octa­hedral coordination environment formed by four carboxyl­ate O atoms from three (4-carboxyl­atophenyl­imino)diacetate ligands and two water mol­ecules. The Zn atoms and Na atoms are linked by (4-carboxyl­atophenyl­imino)diacetate ligands into a three-dimensional framework; the uncoordinated water mol­ecules fill the voids of the skeleton and stabilize it by O—H⋯O hydrogen bonds.

## Related literature

For the synthesis of 2,2′-(4-carboxy­phenyl­azanedi­yl)diacetic acid, see: Young & Sweet (1958 ▶).
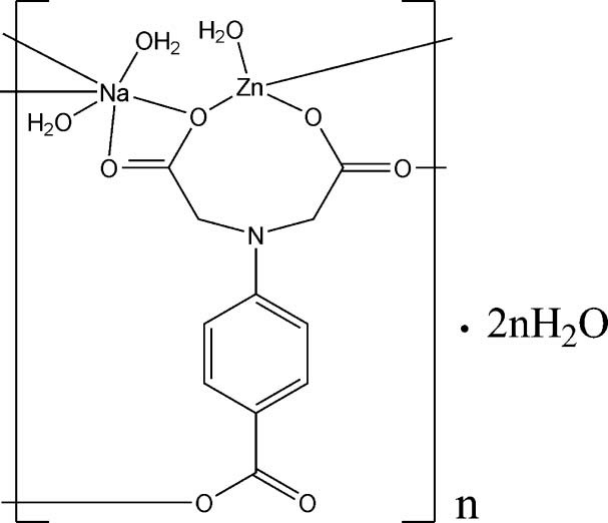


## Experimental

### 

#### Crystal data

[NaZn(C_11_H_8_NO_6_)(H_2_O)_3_]·2H_2_O
*M* *_r_* = 428.62
Triclinic, 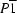

*a* = 7.925 (4) Å
*b* = 8.989 (6) Å
*c* = 11.726 (6) Å
α = 96.28 (3)°
β = 98.63 (2)°
γ = 98.97 (2)°
*V* = 808.1 (8) Å^3^
*Z* = 2
Mo *K*α radiation
μ = 1.61 mm^−1^
*T* = 291 (2) K
0.22 × 0.18 × 0.16 mm

#### Data collection

Rigaku R-AXIS RAPID diffractometer
Absorption correction: multi-scan (*ABSCOR*; Higashi, 1995 ▶) *T*_min_ = 0.718, *T*_max_ = 0.782
8055 measured reflections
3668 independent reflections
3320 reflections with *I* > 2σ(*I*)
*R*_int_ = 0.021

#### Refinement

*R*[*F* ^2^ > 2σ(*F* ^2^)] = 0.026
*wR*(*F* ^2^) = 0.065
*S* = 1.10
3668 reflections
226 parameters
H-atom parameters constrained
Δρ_max_ = 0.28 e Å^−3^
Δρ_min_ = −0.44 e Å^−3^

### 

Data collection: *RAPID-AUTO* (Rigaku, 1998 ▶); cell refinement: *RAPID-AUTO*; data reduction: *CrystalStructure* (Rigaku/MSC, 2002 ▶); program(s) used to solve structure: *SHELXS97* (Sheldrick, 2008 ▶); program(s) used to refine structure: *SHELXL97* (Sheldrick, 2008 ▶); molecular graphics: *SHELXTL* (Sheldrick, 2008 ▶); software used to prepare material for publication: *SHELXL97*.

## Supplementary Material

Crystal structure: contains datablocks global, I. DOI: 10.1107/S1600536808036441/ng2512sup1.cif

Structure factors: contains datablocks I. DOI: 10.1107/S1600536808036441/ng2512Isup2.hkl

Additional supplementary materials:  crystallographic information; 3D view; checkCIF report

## Figures and Tables

**Table 1:** Hydrogen-bond geometry (Å, °)

*D*—H⋯*A*	*D*—H	H⋯*A*	*D*⋯*A*	*D*—H⋯*A*
O7—H10⋯O9^i^	0.85	1.87	2.716 (3)	174
O7—H9⋯O5^ii^	0.85	2.06	2.867 (2)	159
O8—H12⋯O5^iii^	0.85	1.90	2.748 (3)	173
O8—H11⋯O11^i^	0.85	1.97	2.798 (2)	163
O9—H14⋯O1^iv^	0.85	2.07	2.910 (2)	168
O9—H13⋯O8^i^	0.85	1.96	2.801 (2)	172
O10—H17⋯O1^iv^	0.85	1.92	2.762 (3)	174
O11—H15⋯O6^vi^	0.85	2.16	2.949 (3)	154
O11—H16⋯O10	0.85	1.89	2.721 (3)	165

Symmetry codes: (i) 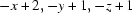; (ii) 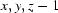; (iii) 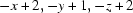; (iv) 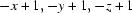; (v) 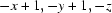; (vi) 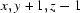.

**Fig. 1.**
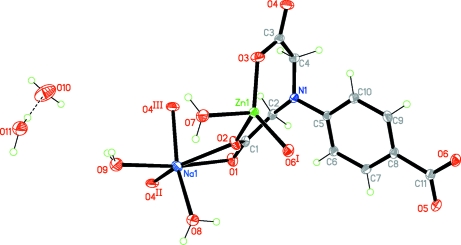
The molecular structure of (I), showing displacement ellipsoids at the 30% probability level for non-H atoms. Dashed lines indicate the hydrogen-bonding interactions [Symmetry code: (I) -x + 2, -y, -z + 2; (II) x, y + 1, z;(III) -x + 1, -y, z + 1].

**Fig. 2.**
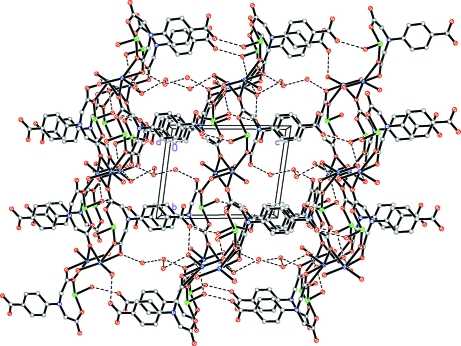
Part of the polymeric structure of (I), showing a three-dimensional framework.Dashed lines indicate the hydrogen-bonding interactions

## supplementary crystallographic information

### Comment

2,2'-(4-Carboxyphenylazanediyl)diacetic acid is a multidentate flexible ligand with versatile binding abilities and capability of participating in hydrongen bonds, thus representing an excellent candidate for the construction of supramolecular complexe. In this paper, we report a novel title compound, (I), which is prepared by 2,2'-(4-carboxyphenylazanediyl)diacetic acid ligand and Zinc dinitrate under neutral aqueous conditions, which forms a three-dimensional framework structure.

The asymmetric unit of (1) consists of one Zn(II) ion, one Na(I) ion, one 4-carboxylatophenylimino)diacetate anion, three coordinated water molecules and two uncoordinated water molecules (Fig. 1). The Zn(II) ion is in a tetrahedral coordination environment, formed by three carboxylate O atoms from two 4-carboxylatophenylimino)diacetate ligands and one water molecules. The Na(I) ion exists in a distorted octahedral configuration with the equatorial plane being defined by the atoms O1, O2, O4^II^ and O8, and with O9 and o4^III^ occupy the axial sites. Each 4-carboxylatophenylimino)diacetate anion bridged two Zn(II) ions and three Na(I) ions to form a three-dimensional supramolecular framework network in which uncoordinated water molecules filled the space of the skelecton and stabilized by O—H···O hydrogen bonds(Fig. 2, Table 1).

### Experimental

2,2'-(4-Carboxyphenylazanediyl)diacetic acid was synthesized by the literature method (Young *et al.*, 1958). The complex (I) was synthesized with znic(II) dinitrate (0.375 g, 2 mmol) and 2,2'-(4-Carboxyphenylazanediyl)diacetic acid (0.253 g, 1 mmol) were dissolved in methanol and the pH was adjusted to about 7 with 0.01*M* sodium hydroxide. Colorless crystals were separated from the filtered solution after several days.

### Refinement

H atoms bound to C atoms were placed in calculated positions and treated as riding on their parent atoms, with C—H = 0.93 Å, 0.97 Å for aromatic and methylene H atoms respectively; *U*_iso_(H) was set to = 1.2*U*_eq_ of the carrier atom. Water H atoms were placed in calculated positions, with O—H=0.85 Å, *U*_iso_(H) = 1.*U*_eq_(O).

### Crystal data

**Table:**

[NaZn(C_11_H_8_NO_6_)(H_2_O)_3_]·2H_2_O	*Z* = 2
*M**_r_* = 428.62	*F*_000_ = 440
Triclinic, *P*1	*D*_x_ = 1.762 Mg m^−^^3^
Hall symbol: -P 1	Mo *K*α radiation λ = 0.71073 Å
*a* = 7.925 (4) Å	Cell parameters from 7215 reflections
*b* = 8.989 (6) Å	θ = 3.1–27.5º
*c* = 11.726 (6) Å	µ = 1.61 mm^−^^1^
α = 96.28 (3)º	*T* = 291 (2) K
β = 98.63 (2)º	Block, colorless
γ = 98.97 (2)º	0.22 × 0.18 × 0.16 mm
*V* = 808.1 (8) Å^3^	

### Data collection

**Table:**

Rigaku R-AXIS RAPID diffractometer	3668 independent reflections
Radiation source: fine-focus sealed tube	3320 reflections with *I* > 2σ(*I*)
Monochromator: graphite	*R*_int_ = 0.021
*T* = 291(2) K	θ_max_ = 27.5º
ω scans	θ_min_ = 3.1º
Absorption correction: Multi-scan(ABSCOR; Higashi, 1995)	*h* = −10→9
*T*_min_ = 0.718, *T*_max_ = 0.782	*k* = −11→11
8055 measured reflections	*l* = −14→15

### Refinement

**Table:**

Refinement on *F*^2^	Secondary atom site location: difference Fourier map
Least-squares matrix: full	Hydrogen site location: inferred from neighbouring sites
*R*[*F*^2^ > 2σ(*F*^2^)] = 0.026	H-atom parameters constrained
*wR*(*F*^2^) = 0.065	*w* = 1/[σ^2^(*F*_o_^2^) + (0.0288*P*)^2^ + 0.3237*P*] where *P* = (*F*_o_^2^ + 2*F*_c_^2^)/3
*S* = 1.11	(Δ/σ)_max_ = 0.001
3668 reflections	Δρ_max_ = 0.28 e Å^−^^3^
226 parameters	Δρ_min_ = −0.44 e Å^−^^3^
Primary atom site location: structure-invariant direct methods	Extinction correction: none

### Special details

**Table:**

Geometry. All e.s.d.'s (except the e.s.d. in the dihedral angle between two l.s. planes) are estimated using the full covariance matrix. The cell e.s.d.'s are taken into account individually in the estimation of e.s.d.'s in distances, angles and torsion angles; correlations between e.s.d.'s in cell parameters are only used when they are defined by crystal symmetry. An approximate (isotropic) treatment of cell e.s.d.'s is used for estimating e.s.d.'s involving l.s. planes.
Refinement. Refinement of *F*^2^ against ALL reflections. The weighted *R*-factor *wR* and goodness of fit *S* are based on *F*^2^, conventional *R*-factors *R* are based on *F*, with *F* set to zero for negative *F*^2^. The threshold expression of *F*^2^ > σ(*F*^2^) is used only for calculating *R*-factors(gt) *etc*. and is not relevant to the choice of reflections for refinement. *R*-factors based on *F*^2^ are statistically about twice as large as those based on *F*, and *R*- factors based on ALL data will be even larger.

### Fractional atomic coordinates and isotropic or equivalent isotropic displacement parameters (Å^2^)

**Table:**

	*x*	*y*	*z*	*U*_iso_*/*U*_eq_	
C1	0.5758 (2)	0.2908 (2)	0.70951 (15)	0.0234 (4)	
C2	0.4639 (2)	0.1504 (2)	0.73561 (15)	0.0228 (4)	
H1	0.4196	0.1764	0.8066	0.027*	
H2	0.3653	0.1200	0.6730	0.027*	
C3	0.5765 (2)	−0.1842 (2)	0.59130 (15)	0.0225 (4)	
C4	0.4695 (2)	−0.1219 (2)	0.67581 (15)	0.0249 (4)	
H8	0.3614	−0.1063	0.6318	0.030*	
H7	0.4413	−0.1979	0.7259	0.030*	
C5	0.6392 (2)	0.00980 (19)	0.86167 (14)	0.0189 (3)	
C6	0.6706 (2)	0.13196 (19)	0.95095 (15)	0.0223 (3)	
H3	0.6319	0.2219	0.9372	0.027*	
C7	0.7589 (2)	0.1201 (2)	1.05973 (15)	0.0232 (4)	
H4	0.7757	0.2014	1.1189	0.028*	
C8	0.8230 (2)	−0.0111 (2)	1.08227 (14)	0.0210 (3)	
C9	0.7931 (2)	−0.1318 (2)	0.99287 (16)	0.0247 (4)	
H5	0.8350	−0.2205	1.0063	0.030*	
C10	0.7025 (2)	−0.1227 (2)	0.88446 (15)	0.0237 (4)	
H6	0.6834	−0.2053	0.8261	0.028*	
C11	0.9258 (2)	−0.0177 (2)	1.19854 (15)	0.0223 (4)	
N1	0.5524 (2)	0.02088 (16)	0.74928 (12)	0.0218 (3)	
Na1	0.70264 (10)	0.49576 (8)	0.57052 (7)	0.02913 (17)	
O1	0.52052 (19)	0.41253 (16)	0.71895 (12)	0.0337 (3)	
O2	0.71665 (17)	0.27959 (15)	0.67283 (12)	0.0294 (3)	
O3	0.70204 (18)	−0.09667 (16)	0.56440 (12)	0.0310 (3)	
O4	0.53062 (19)	−0.31883 (15)	0.54829 (12)	0.0340 (3)	
O5	0.9395 (2)	0.08672 (16)	1.28035 (11)	0.0331 (3)	
O6	1.00014 (17)	−0.13340 (15)	1.21007 (11)	0.0272 (3)	
O7	0.9495 (2)	0.17005 (17)	0.52465 (12)	0.0381 (3)	
H9	0.9528	0.1236	0.4581	0.057*	
H10	1.0276	0.2489	0.5443	0.057*	
O8	0.9626 (2)	0.60310 (17)	0.69752 (13)	0.0380 (3)	
H12	0.9924	0.6993	0.7101	0.057*	
H11	0.9728	0.5703	0.7630	0.057*	
O9	0.81948 (19)	0.56713 (17)	0.40602 (13)	0.0371 (3)	
H13	0.8786	0.5073	0.3756	0.056*	
H14	0.7250	0.5671	0.3609	0.056*	
O10	0.6141 (3)	0.4735 (3)	0.09255 (19)	0.0770 (7)	
H15	1.0046	0.6401	0.1287	0.116*	
H16	0.8541	0.5354	0.0951	0.116*	
O11	0.9641 (2)	0.54605 (19)	0.10841 (14)	0.0459 (4)	
H17	0.5653	0.5064	0.1476	0.069*	
H18	0.5412	0.4016	0.0505	0.069*	
Zn1	0.81967 (3)	0.09629 (2)	0.651229 (17)	0.02072 (7)	

### Atomic displacement parameters (Å^2^)

**Table:**

	*U*^11^	*U*^22^	*U*^33^	*U*^12^	*U*^13^	*U*^23^
C1	0.0252 (9)	0.0272 (9)	0.0201 (8)	0.0090 (7)	0.0039 (7)	0.0070 (7)
C2	0.0215 (8)	0.0277 (9)	0.0206 (8)	0.0076 (7)	0.0028 (7)	0.0054 (7)
C3	0.0231 (9)	0.0225 (8)	0.0186 (8)	0.0043 (7)	−0.0058 (7)	0.0019 (6)
C4	0.0249 (9)	0.0242 (9)	0.0217 (8)	−0.0020 (7)	0.0002 (7)	0.0000 (7)
C5	0.0194 (8)	0.0215 (8)	0.0163 (7)	0.0018 (6)	0.0045 (6)	0.0046 (6)
C6	0.0267 (9)	0.0198 (8)	0.0210 (8)	0.0070 (7)	0.0020 (7)	0.0037 (6)
C7	0.0248 (9)	0.0239 (9)	0.0198 (8)	0.0040 (7)	0.0018 (7)	0.0005 (7)
C8	0.0181 (8)	0.0269 (9)	0.0189 (8)	0.0032 (7)	0.0043 (7)	0.0070 (7)
C9	0.0289 (9)	0.0217 (8)	0.0260 (9)	0.0080 (7)	0.0058 (8)	0.0077 (7)
C10	0.0319 (9)	0.0209 (8)	0.0193 (8)	0.0073 (7)	0.0054 (7)	0.0013 (6)
C11	0.0172 (8)	0.0266 (9)	0.0226 (8)	−0.0008 (7)	0.0031 (7)	0.0085 (7)
N1	0.0267 (8)	0.0208 (7)	0.0165 (6)	0.0044 (6)	−0.0005 (6)	0.0019 (5)
Na1	0.0296 (4)	0.0250 (4)	0.0319 (4)	0.0013 (3)	0.0037 (3)	0.0070 (3)
O1	0.0411 (8)	0.0296 (7)	0.0385 (8)	0.0181 (6)	0.0157 (7)	0.0107 (6)
O2	0.0273 (7)	0.0265 (7)	0.0412 (8)	0.0108 (5)	0.0144 (6)	0.0143 (6)
O3	0.0303 (7)	0.0297 (7)	0.0298 (7)	−0.0017 (6)	0.0099 (6)	−0.0067 (5)
O4	0.0383 (8)	0.0212 (7)	0.0369 (8)	0.0023 (6)	−0.0018 (6)	−0.0049 (6)
O5	0.0431 (8)	0.0325 (7)	0.0206 (6)	0.0053 (6)	−0.0041 (6)	0.0043 (5)
O6	0.0240 (6)	0.0305 (7)	0.0269 (6)	0.0076 (5)	−0.0019 (5)	0.0081 (5)
O7	0.0432 (9)	0.0404 (8)	0.0272 (7)	−0.0084 (7)	0.0159 (6)	−0.0012 (6)
O8	0.0471 (9)	0.0291 (7)	0.0329 (7)	0.0003 (6)	−0.0020 (7)	0.0049 (6)
O9	0.0320 (8)	0.0427 (9)	0.0359 (8)	0.0032 (6)	0.0076 (6)	0.0048 (6)
O10	0.0630 (13)	0.0963 (18)	0.0657 (13)	0.0182 (12)	0.0178 (11)	−0.0318 (12)
O11	0.0583 (10)	0.0381 (9)	0.0392 (8)	0.0073 (8)	0.0024 (8)	0.0052 (7)
Zn1	0.02166 (11)	0.02060 (11)	0.01927 (10)	0.00437 (7)	0.00116 (8)	0.00223 (7)

### Geometric parameters (Å, °)

**Table:**

C1—O1	1.240 (2)	Na1—O4^i^	2.3239 (19)
C1—O2	1.269 (2)	Na1—O8	2.353 (2)
C1—C2	1.513 (3)	Na1—O9	2.3687 (19)
C2—N1	1.461 (2)	Na1—O4^ii^	2.3901 (19)
C2—H1	0.9700	Na1—O2	2.4009 (19)
C2—H2	0.9700	Na1—O1	2.5245 (19)
C3—O4	1.235 (2)	Na1—Na1^iii^	3.401 (2)
C3—O3	1.270 (2)	Na1—H14	2.6320
C3—C4	1.514 (3)	O2—Zn1	1.9584 (16)
C4—N1	1.465 (2)	O3—Zn1	1.9336 (17)
C4—H8	0.9700	O4—Na1^iv^	2.3239 (19)
C4—H7	0.9700	O4—Na1^ii^	2.3901 (19)
C5—C6	1.396 (2)	O6—Zn1^v^	1.9574 (15)
C5—C10	1.399 (2)	O7—Zn1	2.0400 (16)
C5—N1	1.413 (2)	O7—H9	0.8500
C6—C7	1.383 (2)	O7—H10	0.8500
C6—H3	0.9300	O8—H12	0.8500
C7—C8	1.391 (3)	O8—H11	0.8500
C7—H4	0.9300	O9—H13	0.8500
C8—C9	1.389 (3)	O9—H14	0.8500
C8—C11	1.492 (2)	O10—H17	0.8498
C9—C10	1.380 (3)	O10—H18	0.8504
C9—H5	0.9300	O11—H15	0.8504
C10—H6	0.9300	O11—H16	0.8499
C11—O5	1.246 (2)	Zn1—O6^v^	1.9574 (15)
C11—O6	1.283 (2)		
			
O1—C1—O2	122.18 (17)	O4^i^—Na1—O2	138.34 (6)
O1—C1—C2	117.99 (17)	O8—Na1—O2	84.49 (6)
O2—C1—C2	119.71 (16)	O9—Na1—O2	132.16 (6)
N1—C2—C1	114.73 (15)	O4^ii^—Na1—O2	80.48 (7)
N1—C2—H1	108.6	O4^i^—Na1—O1	85.66 (6)
C1—C2—H1	108.6	O8—Na1—O1	98.94 (7)
N1—C2—H2	108.6	O9—Na1—O1	168.52 (6)
C1—C2—H2	108.6	O4^ii^—Na1—O1	78.25 (7)
H1—C2—H2	107.6	O2—Na1—O1	52.90 (5)
O4—C3—O3	123.36 (18)	O4^i^—Na1—Na1^iii^	44.60 (5)
O4—C3—C4	116.88 (17)	O8—Na1—Na1^iii^	153.40 (6)
O3—C3—C4	119.70 (16)	O9—Na1—Na1^iii^	89.94 (6)
N1—C4—C3	115.30 (15)	O4^ii^—Na1—Na1^iii^	43.06 (5)
N1—C4—H8	108.4	O2—Na1—Na1^iii^	113.22 (5)
C3—C4—H8	108.4	O1—Na1—Na1^iii^	78.77 (6)
N1—C4—H7	108.4	O4^i^—Na1—H14	75.5
C3—C4—H7	108.4	O8—Na1—H14	109.2
H8—C4—H7	107.5	O9—Na1—H14	18.6
C6—C5—C10	118.34 (15)	O4^ii^—Na1—H14	77.8
C6—C5—N1	121.25 (15)	O2—Na1—H14	138.5
C10—C5—N1	120.35 (15)	O1—Na1—H14	149.9
C7—C6—C5	120.44 (16)	Na1^iii^—Na1—H14	71.4
C7—C6—H3	119.8	C1—O1—Na1	87.44 (12)
C5—C6—H3	119.8	C1—O2—Zn1	127.16 (12)
C6—C7—C8	121.26 (17)	C1—O2—Na1	92.39 (10)
C6—C7—H4	119.4	Zn1—O2—Na1	134.30 (7)
C8—C7—H4	119.4	C3—O3—Zn1	126.24 (12)
C9—C8—C7	118.10 (16)	C3—O4—Na1^iv^	124.19 (12)
C9—C8—C11	121.76 (16)	C3—O4—Na1^ii^	143.46 (12)
C7—C8—C11	120.11 (16)	Na1^iv^—O4—Na1^ii^	92.34 (7)
C10—C9—C8	121.31 (16)	C11—O6—Zn1^v^	111.56 (12)
C10—C9—H5	119.3	Zn1—O7—H9	128.8
C8—C9—H5	119.3	Zn1—O7—H10	117.0
C9—C10—C5	120.52 (16)	H9—O7—H10	112.8
C9—C10—H6	119.7	Na1—O8—H12	119.1
C5—C10—H6	119.7	Na1—O8—H11	114.2
O5—C11—O6	121.83 (16)	H12—O8—H11	107.7
O5—C11—C8	120.69 (16)	Na1—O9—H13	116.7
O6—C11—C8	117.47 (16)	Na1—O9—H14	98.5
C5—N1—C2	117.69 (14)	H13—O9—H14	111.0
C5—N1—C4	116.93 (14)	H17—O10—H18	106.8
C2—N1—C4	115.93 (14)	H15—O11—H16	107.9
O4^i^—Na1—O8	108.97 (7)	O3—Zn1—O6^v^	128.18 (6)
O4^i^—Na1—O9	87.74 (7)	O3—Zn1—O2	125.40 (7)
O8—Na1—O9	92.08 (7)	O6^v^—Zn1—O2	100.31 (7)
O4^i^—Na1—O4^ii^	87.66 (7)	O3—Zn1—O7	97.01 (7)
O8—Na1—O4^ii^	162.99 (6)	O6^v^—Zn1—O7	103.35 (7)
O9—Na1—O4^ii^	92.12 (7)	O2—Zn1—O7	93.95 (7)

Symmetry codes: (i) *x*, *y*+1, *z*; (ii) −*x*+1, −*y*, −*z*+1; (iii) −*x*+1, −*y*+1, −*z*+1; (iv) *x*, *y*−1, *z*; (v) −*x*+2, −*y*, −*z*+2.

### Hydrogen-bond geometry (Å, °)

**Table:**

*D*—H···*A*	*D*—H	H···*A*	*D*···*A*	*D*—H···*A*
O7—H10···O9^vi^	0.85	1.87	2.716 (3)	174
O7—H9···O5^vii^	0.85	2.06	2.867 (2)	159
O8—H12···O5^viii^	0.85	1.90	2.748 (3)	173
O8—H11···O11^vi^	0.85	1.97	2.798 (2)	163
O9—H14···O1^iii^	0.85	2.07	2.910 (2)	168
O9—H13···O8^vi^	0.85	1.96	2.801 (2)	172
O10—H17···O1^iii^	0.85	1.92	2.762 (3)	174
O10—H18···O10^ix^	0.85	2.41	2.744 (5)	104
O11—H15···O6^x^	0.85	2.16	2.949 (3)	154
O11—H16···O10	0.85	1.89	2.721 (3)	165

Symmetry codes: (vi) −*x*+2, −*y*+1, −*z*+1; (vii) *x*, *y*, *z*−1; (viii) −*x*+2, −*y*+1, −*z*+2; (iii) −*x*+1, −*y*+1, −*z*+1; (ix) −*x*+1, −*y*+1, −*z*; (x) *x*, *y*+1, *z*−1.
